# Spider Origami: Folding Principle of Jumping Spider Leg Joints for Bioinspired Fluidic Actuators

**DOI:** 10.1002/advs.202003890

**Published:** 2021-01-21

**Authors:** Chantal Göttler, Karin Elflein, Roland Siegwart, Metin Sitti

**Affiliations:** ^1^ Physical Intelligence Department Max Planck Institute for Intelligent Systems Stuttgart 70569 Germany; ^2^ Autonomous Systems Laboratory ETH Zurich Zürich 8092 Switzerland; ^3^ Institute for Biomedical Engineering ETH Zurich Zürich 8092 Switzerland

**Keywords:** bioinspired robotics, fluidic actuators, hydraulics, jumping spiders

## Abstract

Jumping spiders (*Phidippus regius*) are known for their ability to traverse various terrains and have targeted jumps within the fraction of a second to catch flying preys. Different from humans and insects, spiders use muscles to flex their legs, and hydraulic actuation for extension. By pressurizing their inner body fluid, they can achieve fast leg extensions for running and jumping. Here, the working principle of the articular membrane covering the spider leg joint pit is investigated. This membrane is highly involved in walking, grasping, and jumping motions. Hardness and stiffness of the articular membrane is studied using nanoindentation tests and preparation methods for scanning electron microscopy and histology are developed to give detailed information about the inner and outer structure of the leg joint and its membrane. Inspired by the stroller umbrella‐like folding mechanism of the articular membrane, a robust thermoplastic polyurethane‐based rotary semifluidic actuator is demonstrated, which shows increased durability, achieves working angles over 120°, produces high torques which allows lifts over 100 times of its own weight and jumping abilities. The developed actuator can be used for future grasping tasks, safe human–robot interactions and multilocomotion ground robot applications, and it can shed light into spider locomotion‐related questions.

## Graphical abstract

This work investigates the folding behavior of the articular membrane covering the jumping spider leg joint pit. This membrane is essential for the semihydraulic actuation of spiders. The mechanical properties and detailed microscopy information about the inner and outer membrane structures are provided, and a spider‐inspired mechanism with a rotary semifluidic actuator, which can lift 100 times its weight and enable jumping, is presented.

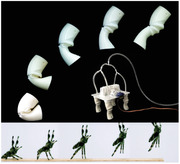

Traditional fluidic actuators, such as hydraulic pistons, are almost irreplaceable for many of today's mechanical, robotic, and automation applications due to their powerful and fast motions. To withhold high pressure, fast motions, and heavy loads, usually metal‐based traditional fluidic actuators are used, which are often heavy and bulky. With increasing interest in soft robotics and the importance of safe human–robot interaction, soft and lightweight fluidic actuators have been proposed over the last decades.^[^ ^1^ ^]^ Such soft fluidic actuators have enabled self‐adaptable soft grippers that can grasp various complex objects, shapes and forms robustly.^[^ ^2^, ^3^, ^4^, ^5^ ^]^ The design and material of such actuators allow almost infinite degrees of motion freedom and compliance on the one hand, which can lead to hysteresis and other nonlinearities that would require nontrivial approaches for precise control on the other hand.^[^ ^6^ ^]^

A promising inspiration for hydraulic or semihydraulic actuators is the locomotion mechanism of spiders.^[^ ^7^, ^8^ ^]^ While most arthropods use muscles or elastic proteins, such as resilin, to achieve different types of locomotion modes, arachnids, especially spiders, use a combination of hydraulics and muscles,^[^ ^9^ ^]^ for walking, climbing, and even jumping. Two of their main leg joints (femur‐patella and tibia‐metatarsus) (**Figure** **1**A,B), crucially involved in grasping and jumping, are lacking extensor muscles. Instead, muscles in the front body part (prosoma)^[^ ^9^, ^10^, ^11^, ^12^ ^]^ increase the pressure of the body fluid (hemolymph) to 60 kPa^[^ ^13^ ^]^ or even 130 kPa^[^ ^10^ ^]^, resulting in leg joint extension. In the 1980s, Blickhan and Barth investigated the spider joint as an inspiration for robotic applications,^[^ ^10^ ^]^ opening the field of spider‐inspired actuators.^[^ ^14^, ^15^, ^16^, ^17^ ^]^ Notably, the folding of a thin membrane covering the leg joint pit played a key role in the adaption of the hydraulic principle. Blickhan and Barth have described it as a bellow‐like folded, anisotropic articular membrane.^[^ ^10^ ^]^ Although the inspiration has been around for almost half a century and biological research on the locomotion of spiders can be traced back to 1910, we still do not fully understand the spider leg structure and morphology for the given locomotion mode^[^ ^18^ ^]^ and hydraulic actuators performing as graceful, versatile and powerful as spiders are not available yet. The goal of this work is to provide a better insight into the working principle of the articular membrane and use this principle to design a jumping spider‐inspired robotic leg. As detailed images and videos of the working principle are lacking in the literature, we developed new methods to provide high‐resolution optical images and recordings of the femur‐patella joint showing how the folding mechanism works and how it differs from bellow mechanisms frequently used in the robotics community.

**Figure 1:**
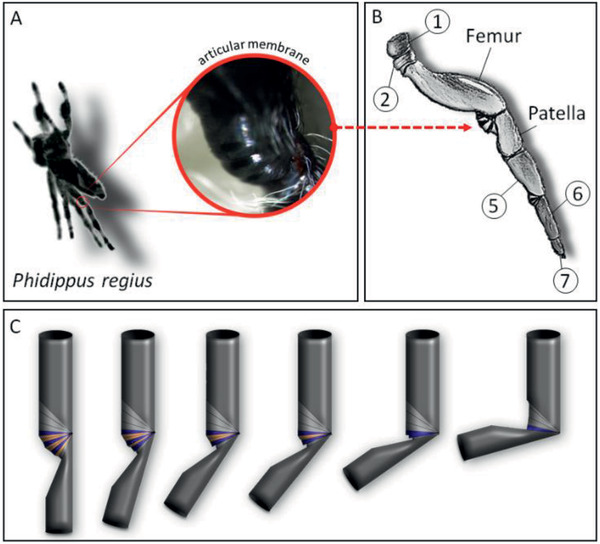
Articular membrane between the femur patella joint in a jumping spider. The femur patella joint of spiders plays a key role in their locomotion (Movie S1, Supporting Information). A) The screenshot of a high‐speed video recorded jump shows the extension of the femur‐patella joint of the studied 2–3 cm large jumping spider *Phidippus regius*. B) Spider legs are divided into seven leg segments: coxa 1), trochanter 2), femur 3), patella 4), tibia 5), metatarsus 6), and tarsus 7). The articular or arthrodial membrane (red circle) is a thin sheet of exoskeleton spanned between the femur‐patella joint. A similar membrane can be found between the tibia‐metatarsus joint. In *Phidippus regius*, the membrane shows a zebra‐like patterning. The difference in coloration is a result of a difference in translucency as the membrane itself is transparent, but the layer below (epithelial cells) is responsible for the coloration. C) The black‐appearing hoops (purple) seem to be stiffer than the transparent (orange) part of the membrane and slide into each other during flexion as shown in this animation.

Furthermore, we analyzed the anisotropy of the membrane, which seems to be a key element of the mechanism by testing the material properties of the membrane. Finally, we developed a fluidic prototype device, which uses similar folding principles as the jumping spider to test the advantages of this new design. The prototype device reported in this work may further serve as a testing platform for biological hypotheses, which cannot be easily tested on the real spider and it could also help to evaluate which aspects of the locomotion of spiders can be useful in bioinspired robotic implementations.

The articular (or arthrodial) membrane plays an essential role in the locomotion of spiders. Although it is just a thin sheet of exoskeleton (cuticle) spanned between the femur and patella segment of spiders (Figure 1B), it enables high working angles (100°–160°^[^ ^13^, ^19^, ^20^ ^]^) and it is part of the hydraulic extension mechanism important for jumping (Figure 1A). The membrane of adult jumping spiders (*Phidippus regius*) span an area of 2–3 mm^2^ with a thickness of around 50 µm. It shows a zebra‐stripe‐like pattern alternating between transparent and black stripes (Figure 1C). During flexion, the transparent section folds underneath the black part (Figure 1D; and Movie S1, Supporting Information), resembling the folding of a stroller sun shade, with the difference that the black rings show a larger width, have a change in diameter and slide into each other like the inner ring of an ice‐cream scoop. These observations lead to the assumption that the black rings show stiffer behavior than the transparent section, supporting the folding and unfolding of the joint. The membrane itself appears to be nonetheless flexible, although it does not expand in the way a balloon would do, but rather unfolds similar to a thin plastic bag when inflated.

While a stiffness difference between the hoops and the rest of the membrane has been proposed before,^[^ ^7^, ^21^ ^]^ it has never been experimentally verified. To prove the concept of the stiffness variance, the articular membrane of the femur‐patella joint was dissected, and different methods were used to visualize the parts that may influence the mechanical properties. Furthermore, nanoindentation was performed on the different parts of the membrane to collect experimental data for hardness and stiffness.

The articular membrane is attached to the arcute sclerite, forming a small chamber where the body fluid acts on for extension (**Figure** **2**A). The cuticle of the articular membrane differs from the general exoskeleton of the leg. Reports show a Young's modulus of around 18 GPa^[^ ^10^ ^]^ for leg segments, due to a highly sclerotized and stiff exocuticle, which is lacking in the articular membrane. Instead, the membrane is formed by a thin epicuticle and a large endocuticle of unidirectional wavy layers of chitin fibers (Figure 2B,C), indicating a fabric‐like soft cuticle.^[^ ^22^ ^]^ Histological cuts indicate thicknesses from 30 µm at the transparent part to 60 µm at the black part (Figure 2B). Since the two areas cannot be easily distinguished in the histological cuts and the membrane tends to deform during sample preparation, the thickness can only be matched roughly to the areas. The dissection also reveals that the zebra‐stripe coloration (Figure 1A) is an effect of translucence differences throughout the articular membrane. The black color is provided by a layer below the whole exoskeleton, the epithelial cells (Figure 2D), which are important in the formation and secretion of cuticle and are known to have pigment corns embedded, giving spiders their distinct colorations.^[^ ^19^ ^]^ The articular membrane itself is transparent. Under the scanning electron microscope (SEM) (**Figure** **3**B,C), the black hoops show line‐patterns, while the transparent part connecting the hoops show dot‐patterns. To avoid confusion as transparency appearance could change depending on the microscope type and instrument used, the two different areas will be from now on labeled as the line area (l) (former: black hoops) and dot area (d) (former: transparent part) according to the described microstructures covering the different sections (Figure 3B). A 10% potassium hydroxide (KOH) treatment of the exoskeleton indicates that these microstructures are part of the epicuticle, the outermost layer of the articular membrane, and probably formed by proteins as they are washed away from the membrane, leaving a transparent, chitin fibered, nonprotein containing construct behind, showing the fabric‐like base material of the articular membrane (Figure S2, Supporting Information).

**Figure 2:**
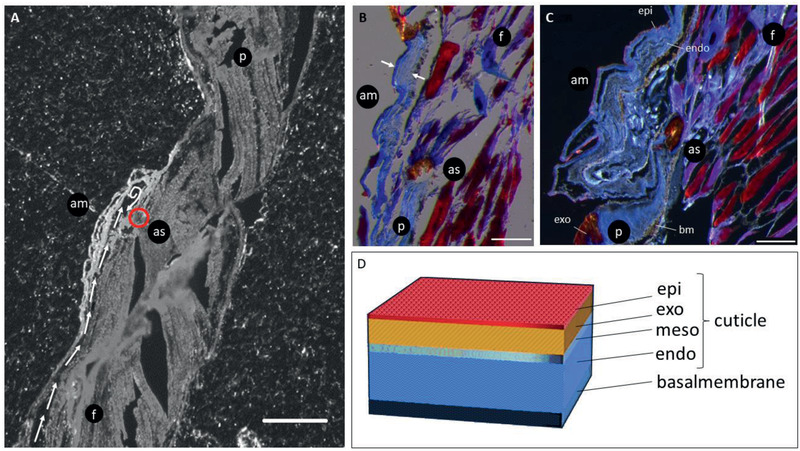
Histological dissection of the femur‐patella joint. A) The articular membrane (am) is attached to the patella (p) segment via arcute sclerite (as), which is a sclerotized, arched piece of exoskeleton and serves as an attachment point for the inner flexion muscles (stained in purple to red color). The hemolymph flows into the chamber from the femur (f) to the patella direction (marked as white arrows). Scale bar: 200 µm. B) The membrane has a thickness of 30–60 µm. The thicker appearing part (marked between the two white arrows can be assigned to the black hoops. Scale bar: 100 µm. C) The membrane is divided into several laminar layers, it shows a thin epicuticular, and a large area of endocuticle, followed by a fragile basal membrane, which easily separates, making the preparation challenging. The membrane lacks a brown appearing exocuticle which could be found, e.g., in the femur or patella segment. Scale bar: 100 µm. D) The spider exoskeleton can consist of four different layers. The outer, waxy epicuticle, the very stiff, sclerotized exocuticle followed by the mesocuticle and the large and soft endocuticle part stained in blue. The basal membrane, formed by epithelial cells underneath the cuticle, consists of pigment corns, which give the exoskeleton its distinct colors.

**Figure 3:**
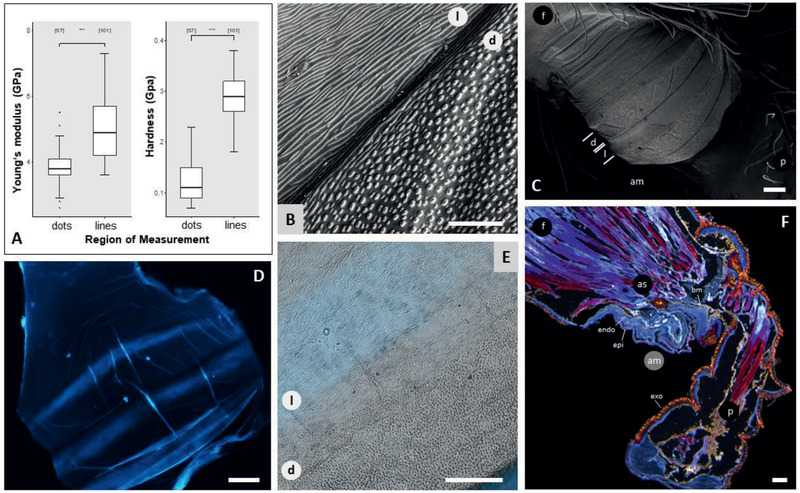
Stiffness variation in the articular membrane. A) Nanoindentation experiments show a significant difference in the Young's modulus and hardness between the dotted (*N* = 57) and line (*N* = 101) areas on the articular membrane. Statistical analysis (Wilcoxon test, see Table S1, Supporting Information) show that the areas differ significantly in their stiffness and hardness with *p*‐values < 0.001 (marked by ***). B) The areas are assigned according to the microstructures covering the surface (epicuticle) of the membrane. Scale bar: 10 µm. C) The SEM image shows an inflated articular membrane, which shows three distinct hoops that are covered with longer line structures and in between brighter appearing areas, covered with dots. Scale bar: 100 µm. D) The resilin quick test shows blue autofluorescence on the line area sections (contrast increased during the post processing), indicating a resilin existence. Scale bar: 50 µm. E) However, the light blue coloration of the fluorescence image (no postprocessing) emphasizes the assumption that not resilin but a higher sclerotization is the cause of the fluorescence. Scale bar: 50 µm. F) The histological cut shows the stiff and highly sclerotized brown exocuticle on the femur and patella leg segment, which is lacking on the articular membrane. Instead, the articular membrane shows laminar layers of endocuticle, giving the articular membrane a softer characteristic compared to the rest of the leg. Scale bar: 100 µm.

During dissection, the articular membrane appears to be flexible and stretchable to some extent (Movie S1, Supporting Information), reminiscent of a very tiny piece of cling film. This observation has brought up the question whether the common elastic protein, resilin, might be embedded into the articular membrane influencing its mechanical properties. Resilin can be found in many insect body parts involved in fast motions (dragonfly wings, locust jumping legs).^[^ ^23^ ^]^ The literature—to our knowledge—does not provide any information about resilin in the articular membranes of spiders and resilin has not been found in spiders so far.^[^ ^24^ ^]^ Therefore, the quick indirect test for resilin was conducted. Strong blue autofluorescence under UV light is an indication for resilin.^[^ ^25^ ^]^ Only the line area of the articular membrane shows a blue response (Figure 3D). However, the observed more light‐bluish color can also be attributed to the epicuticle (Figure 3E).^[^ ^24^ ^]^ Nanoindentation results (Figure 3A; and Table S1, Supporting Information) support the hypothesis of a difference in the mechanical properties between the dot and line areas, showing a mean Young's modulus (*E*) of 3.8 GPa for the dot and 5.0 GPa for the line area. Furthermore, the hardness (*H*) appears to be more than twice as high for the line area than the dot area, with means of 0.12 GPa for the dot and 0.29 GPa for the line area. The general range of the measured Youngs’ modulus of 3–5 GPa coincides with Barth's estimations from 1973,^[^ ^22^ ^]^ where the stiffness properties of the exoskeleton were estimated using different hierarchical structures of the chitin‐microfiber layers (Figure 3F). The difference in mechanical properties could be a result of the thickness difference in different areas.^[^ ^26^ ^]^

The implementation of these observed characteristics of the articular membrane into a semifluidic actuator (SFA) prototype is presented in the following. Main requirements for soft actuators are large deformation, fast motion, and lightweight.^[^ ^5^, ^27^, ^28^ ^]^ Furthermore, low‐cost, reproducibility in fabrication and simple integration into larger systems are of importance. For fluidic actuators, sealing and pressure resistance needs to be guaranteed as well. To fulfil these requirements, various materials and methods have been tested (Table S4, Supporting Information). Finally, thermoplastic polyurethane (TPU) has been considered as the best fitting material. It allows fast prototyping using fused deposition modeling (FDM‐3D printing), shows desirable flexibility properties to achieve the spider‐inspired folding mechanism, as well as resistance to scratch and abrasion. The flexible polymer is also known for its ability to repel a variety of chemical agents including oil and water, which is another desirable feature for hydraulic applications as the hydraulic fluid will not be absorbed by the filament. On top of that, the used TPU filament is recyclable, environment friendly, and food safe.

The actuator was designed to be fully 3D printed without support structures and further cleaning steps (**Figure** **4**A; and Figure S1, Supporting Information). The total finger‐sized 3D‐printed model weighs 6 g and takes 2 h printing time in the fastest printing mode. The thinnest membrane thickness, for given overhang prints (max. 45°), without additional support and still slice‐able for 3D‐printing software (CURA) is 0.45 mm. The prototype consists of an outer membrane spanned between two tubular segments, resembling the femur and patella leg segment of spiders (Figure 4A; and Figure S3, Supporting Information). Hoops of 1 mm thickness with decreasing diameters on top of the membrane support the folding and shape of the membrane. An inner membrane forms a chamber providing a volume of 2.9 cm^3^, so ≈3 mL of water can be filled into the leg in its extended position (Figure 4A‐II). During flexion, the folding mechanism pushes the inner fluid back toward the femur part (Figure 4A‐IV; and Movie S2, Supporting Information). Although the counteracting muscle mechanism is anchored on the inside of the spider leg, the flexion mechanism has been laid to the outside. This simplifies the fabrication in case the wire needs to be exchanged and fits to a modular design principle to extend the design for multilegged or multijoint applications. Fishing lines are chosen as the tendon‐wire for the flexion mechanism, as they are optimized for low stretching at high loads and do not snarl up the way other yarn would do.

**Figure 4:**
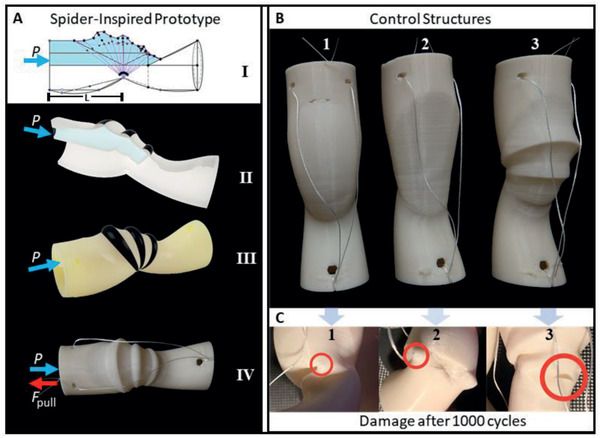
Testing robustness of SFA with and without spider‐inspired design structures. A) The spider‐inspired prototype has a segment length *L* of 3cm (A‐I) and consists of a small chamber (A‐I, blue), which can be filled with water to increase pressure *P* for extension. The dissection shows a chamber which holds up to 3 mL of water (A‐II). The finger‐sized prototype has a length of 6 cm and width of 2 cm. The black marked hoops (A‐III) are 1 mm thick and thin out toward the pivot point. The membrane itself has a thickness of 0.45 mm. The finger‐sized prototype has a length of 6 cm and width of 2 cm. A 4 mm‐diameter hole on the end, serves as tube connector to fill in the water (A‐III). The flexion wires are spanned through two holes at the end of the legs (A‐IV). When pulling the mechanism, the water is pressed out of the chamber. B) Beside the proposed spider‐inspired design, designs without the variance in mechanical properties have been tested as controls (Movie S2, Supporting Information), including a design with a bladder shaped chamber, covering a similar amount in volume but does not show any folding lines nor thicker hoops 1), a version without any shape, but a flat membrane 2), and a design showing folding lines, but no support through thicker hoops 3). C) Durability tests show that the bladder design folds uncontrolled, resulting in crinkles and material failure 1). The flat design results in a sharp fold at the pivot point, creating a hole into the chamber 2). Without the support of stiffer hoops, the membrane folds properly but rips, close to the folding lines 3).

Previously reported spider‐inspired actuators show working angles below 90°.^[^ ^7^, ^16^ ^]^ This limitation could be either due to fabrication limitations^[^ ^15^ ^]^ or torque reduction as a result of the counter‐acting forces by bulging volumes created during the expansion of the hyperelastic and isotropic joint material (e.g., silicone rubber).^[^ ^7^, ^10^, ^16^, ^17^ ^]^ To achieve a larger working angle, the pivot point connection has to be prevented from ripping as the prototype is printed in one step and no hinge pin is used. Therefore, the pivot covers a circle segment of 120°. This results in a thin pivot connection which is, without extensive stretching, minimizing the extension forces created by the connection and reducing the chances of ripping the material after several cycles of movement. To prevent ripping inside, folding lines were integrated to the inner membrane, following the patterns of the outer membrane (Figure 4A‐II).

To investigate the advantage of the proposed design, we compared the folding behavior and durability of legs without the stiffness and thickness variation, but the same pivot point and working angles (Figure 4B). All four models were folded and unfolded for in total 1000 cycles (Figure 4C; and Movie S2, Supporting Information). The “belly control model” (Figure 4B‐1) without the stiffness variation and any folding lines, folds in an uncontrolled way, resulting in undesired crinkles (Figure 4C‐1), which lead to material failure. Less damage occurs in the control model without a large chamber (Figure 4B‐2). The membrane bends like a rubber band but shows a sharp bending wrinkle at the pivot point, causing damage (Figure 4C‐2). The control model with folding lines but without thick hoops (Figure 4B‐3), folds properly, but rips tend to form close to the folding line along the printing layer due to sharp folding edges (Figure 4C‐3). In comparison, the proposed, spider‐inspired design (Figure 4A‐IV) does not show ripping after the same number of cycles. The thick hoops push the membrane along the folding lines, avoiding uncontrolled sharp crinkles.

For experimental characterization, flexion was induced by rotating a water collection reservoir (Figure S1, Supporting Information), resulting in a shortening of the flexion wire (Movie S2, Supporting Information). The reservoir is covered with a soft latex bag inside (Figure S1, Supporting Information), expanding and collecting the displaced water. Pressure, angle, and load have been measured over time during the folding and unfolding of the actuator. The folding process has three phases (phase A, B, and C) while the unfolding process has two phases (phase D and E) (**Figure** **5**). The initial pulling counteracts the elastic forces created by the pivot connection, the inner membrane and the first hoop (phase A). Water is displaced at the end of this phase as folding angles below 20° do not affect the inner leg chamber. Load and pressure constantly increase up to 2.0 N at around 10 kPa. By pulling further, water starts to get displaced into the latex bag of the water reservoir (phase B). Pulling and flexing direction are almost in parallel, resulting in less friction. Measured flexion forces stay almost constant at around 1.5 N for working angles between 30° and 90°. Fluctuations correspond to the stable folding positions (Figure 5A‐I; and Figure S3, Supporting Information). The pressure continuously increases to ≈20 kPa.

**Figure 5:**
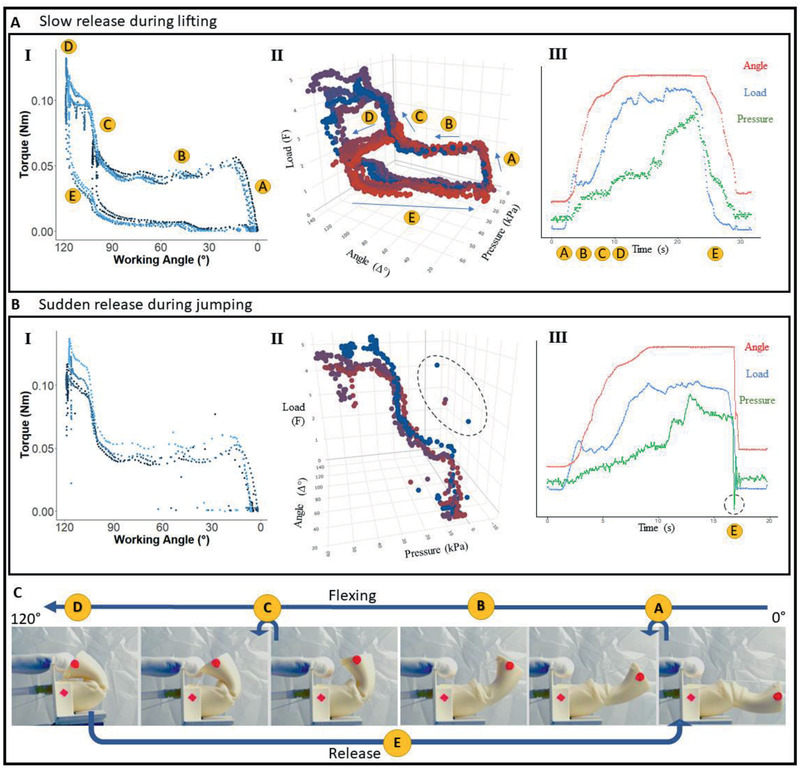
Flexion characteristics of the spider‐inspired prototype. Using a flexion mechanism, the leg was flexed, while the pushed‐out water was collected in a latex bladder simultaneously (Figure S1, Supporting Information). Joint angle, load, and pressure were recorded. Two different release modes followed after the flexion. A) A slow release or extension can be found during lifting and grasping and was therefore tested (*N* = 6). B) During jumping, the pressure is increased inside the chamber, while it is flexed and by a sudden release of the flexion mechanism, the leg snaps back into extended position. To characterize this behavior, the flexion wire was detached, to allow a sudden release (*N* = 4). (A‐II, B‐II) The flexion behavior can be divided into different phases (marked in yellow) (Movie S2, Supporting Information). In the first 20° working angle (phase A), the chamber is not flexed, so there is no pressure increase. When flexing further (phase B), the measured load shows dips for every stable position, but stays almost constant at 1.5 N (≈0.05 Nm torque) (A‐I, B‐I). The flexion forces increase rapidly to 4–5 N, when flexing the leg over 100° (phase C). The pressure constantly increases while flexing the leg, up to 30 kPa. When increasing the pressure manually with a syringe up to 50 kPa (phase D), the load shows almost no increase. Measured increase in load vary from 0.0–1.5N (A‐II). When manually and slowly releasing the leg, the measured load drops down immediately, while pressure decreases constantly with the opening of the chamber (A‐III). When carrying out a sudden release of the leg, the load and pressure drop immediately (dashed circle) (B‐II, B‐III).

When folding the third hoop to achieve the full working angle, a large surface is folded in, creating large counteracting elastic forces (phase C). Measurements show forces of up to 4.5 N at pressures of 30 kPa (Figure 5A‐II). As the pulling direction is almost orthogonal to the folding direction, friction of the flexion wires has to be considered. While keeping the actuator flexed, the pressure was artificially increased by adding water into the system (phase D). The experimental setup allows up to 55 kPa of pressure before tube connections fail. Only a small addition in load of 0.5–1.0 N was achieved (Figure 5A‐I). When reopening the leg continuously, the load drops rapidly down to 1.0 N within 30° of rotation (phase E) (Figure 5A). During a sudden release, by detaching the flexion wire, the leg snaps fully open within 100 ms (Figure 5B). Recorded pressures show negative values, oscillating around zero (Figure 5B‐II,III).

The rotary actuator straightens when increasing the pressure inside the chamber. This movement is supported by the hyperelastic material properties of TPU, leading to immediate extension of the leg when removing any counteracting (antagonistic) forces. For performance predictions, the experimental set up was changed to test at higher pressures (Figure S3, Supporting Information). Short tubes with fixed tube connection were used to allow pressures of 200–300 kPa before tube connection or material fail. To overcome leakages at high pressures due to printing irregularities different methods have been tested (Table S5, Supporting Information). The most promising method was covering the inside of the chamber with a thin latex layer, sealing printing irregularities without influencing the folding performance. As the flexion wire is not connected through the holes during lifting and jumping demonstrations and the position of the wire has a large influence on the measured load, extension created by the material itself at zero pressure was verified by hanging weights to the leg, measuring the minimal weight needed to flex the extended leg into a specific angle and position (Table S6, Supporting Information). Afterward, the leg was folded manually to a given angle, pressurized using a syringe and the resulting extension load was recorded, while keeping the leg flexed. The pressure‐load behavior can be estimated with linear fits (**Figure** **6**A), showing a mean slope of 0.013 corresponding to an effective area of 13 mm^2^ (Table S6, Supporting Information).

**Figure 6:**
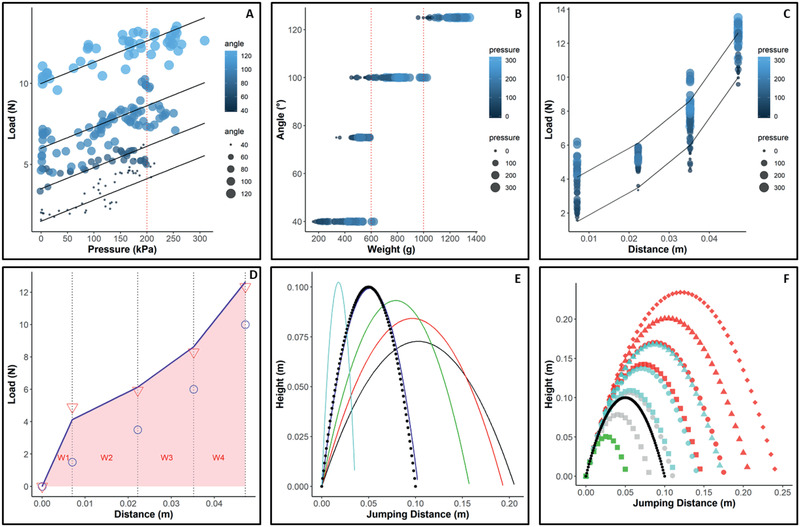
Leg joint extension characteristics. A) For estimating jumping and lifting performances, load and pressure at the stable positions (40°, 75°, 100°, 125°) have been measured by holding the leg in a given position and manually increasing the pressure up to 200 kPa with a syringe. The value of angles was always defined as 0° in extended position and 125° in fully flexed position (*N* = 3, for each position). Loads at zero pressure, reflecting the elastic forces of the material, were verified by the weight needed to flex the leg into a specific position (Table S6, Supporting Information). Linear fits show a factor of 0.013, which would reflect an effective area of 13 mm^2^. B) By transforming the measured load into its corresponding weight, the experimental data predict full lifting of 600 g at 200 kPa. Furthermore, up to 1 kg can be lifted for 20°. C) Load at 200 kPa can be estimated by the linear fits. By transforming the angle into opening distances (Figure S3, Supporting Information), the work generated by a leg can be calculated. D) The work, *W*, can be approximated by the area (light red) below the load‐distance curve and shows a quadratic characteristic (see the Supporting Information). Blue circles show forces created by the elastic material; red triangles show mean of experimental data at pressures (200 kPa +/− 5kPa). E) This was used to estimate the jumping height and jumping distance at launching angles of 45°–85° and different initial flexion angles. F) The flexion angles of front and hind legs are evaluated separately and vary between 75° and 90° (shapes reflecting the flexion angles of front legs, and color of hind legs, with green square corresponding to 90° in both legs). The best jumping performance of the jumping demonstration shows a launching angle of 70°–75° with front legs flexed in 80°–90° and hind legs in 70°–75°, with a jumping height and distance of ≈10 cm (black curve), which fits to the estimated calculations based on the load‐pressure experiments.

The experimental data predict full lifting of 600 g at 200 kPa and 1 kg weight can be lifted for at least 20° (Figure 6B). For jumping height estimation, the opening distance (Movie S2, Supporting Information) was calculated from the corresponding angle value and a segment length of 3 cm (Figure 4A‐I; and Figure S3, Supporting Information). The generated work was estimated from the load measurements at 200 kPa (Figure 6C,D). Flexing and releasing the jumping platform at zero pressure, showed that work generated by the elastic material is necessary to lift the 120 g platform (see next section) from “sitting” back in standing position. Therefore, this work, generated by the elastic material, was subtracted to estimate jumping performances (see the Supporting Information). The maximal useful flexion angle was 90°, as higher flexion angles in this arrangement would have result in the leg segment just hitting the ground. A maximum jumping height of 23 cm and a jumping distance of 24 cm at 200 kPa, with all four legs flexed to 90° and a launching angle of 75° could be predicted (Figure 6E,F). The jumping prediction shows that the jumping performance highly depends on the initial flexed position.

To demonstrate the performance of the actuator prototype (with inner latex layer for sealing), weights up to 2 kg were attached to a vertical actuator and lifted by pressurizing the leg directly with a syringe (**Figure** **7**A). The prototype was able to fully lift 600–800 g (Figure 7B) at 200–250 kPa (Movie S3, Supporting Information). Heavier load, up to 2 kg, could still be lifted to the 90°–100° position, although with higher loads, leakages through tube connection occur and small water droplets start to form on the membrane surface, indicating material failure at pressures above 200 kPa. The reinforced thicker hoops cannot withhold the inner pressure anymore and the chamber starts to blow up like a balloon resulting in less leg extension. By drying the leg, letting the inner water evaporate and refilling the leg with latex milk afterward can help sealing and healing small wounds.

**Figure 7:**
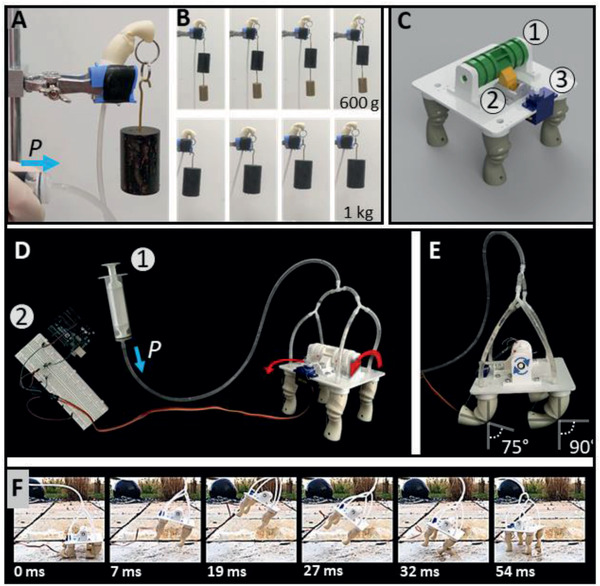
Demonstration of lifting and jumping performance. A) Lifting performance of the SFA was tested by attaching up to 2 kg of weight (Movie S3, Supporting Information) on the prototype and increasing the hydraulic pressure *P* with a syringe to cause extension. B) Weights of 600–800 g could be lifted fully, while heavier load could only be lifted for 90° as predicted in the estimations (Figure 6). C) The robotic jumping platform consists of a plate where four legs are attached to. The legs are flexed by a ratchet (1, green), which is released when the servo motor (3, blue) pulls on the stopper (2, yellow). Scale bar: 3 cm. D) Tubes filled with water are connected to each other to one single input tube, which is connected to a syringe 1) for pressure increase. The legs are connected to a ratchet via wires and flexed manually. The release mechanism via stepper motor is controlled by an Arduino Uno (2). E) When turning the ratchet, the legs get flexed into angles between 75° and 90°. Further flexing would result in the leg segment hitting the ground without any further impact on the jumping performance. F) Video snapshots (Movie S3, Supporting Information) show flexion angles of 75°–90° during the robot jumping tests. The best jumping performance resulted in around 10 cm jumping height.

For jumping demonstration, four legs were attached to a jumping platform (Figure 7C). The flexion strings were attached to a 3D‐printed ratchet, so that all four legs could be flexed simultaneously. The legs were connected via tubes to a syringe (Figure 7D). A small servo motor served as the release mechanism, controlled by an Arduino Uno (Figure 7D). The whole construct filled with water weighed 120 g. Flexion angles of 80°–90° in the two front legs and 75°–80° in the hind legs were used for jumping demonstrations (Figure 7E). From flexed position into extension a maximal opening distance of 3 cm was covered. The best jumping performance (Figure 7F; and Movie S3, Supporting Information) fit to the estimated jumping height of 9–10 and 10–11 cm jumping distance for flexion angles between 75° and 90° and at 75° launching angle (Figure 6E,F).

In this work, we successfully developed new preparation and microscopy methods to provide a closer look on the femur‐patella joint of the jumping spider *Phidippus regius* and detailed information about its working principle. We implemented this principle into a flexible semifluidic actuator and demonstrated that the proposed design allows longer durability, can lift large weights compared to its own size and weight and is capable of jumping. The experimental procedures and outcomes including the biological study, the bioinspired principle and the learnings from the implementation are elucidated in the following discussions.

The recorded videos reveal that the folding mechanism does not fully resemble a bellow as proposed by Blickhan and Barth.^[^ ^10^ ^]^ Instead, our observations coincide with Manton's drawings from 1958,^[^ ^21^ ^]^ mentioning five sclerotized (stiffer cuticle), amber‐colored hoops, shuffling on top of each other. Unfortunately, this observation fell into oblivion, although her drawings and description of the membrane structure and folding were represented in detailed. The main difference between our observation and Manton's description is that the jumping spider *Phidippus regius* has only three, instead of five, black‐colored stiffer‐appearing rings sliding into each other and reinforcing the membrane as proposed by Landkammer.^[^ ^7^ ^]^ SEM and histological sample preparation turned out to be challenging as dead spiders tend to have flexed legs, forming the characteristic “mummy position.” The legs deflate and when reopening the joints manually during preparation, the articular membrane is partially deflated and does not show its natural (stroller sunshade) form under the inflated condition. As traditional fixation methods do not consider this problem, we developed a preparation and fixation procedure to visualize inflated spider leg samples for SEM and histology (see the Experimental Section). In the resulted high‐resolution images, we discovered alternating microstructure patterns on the articular membrane, which have not been reported in the literature so far and seem to differ among species. This patterning might reveal evolutionary adaptions to different locomotion behaviors.

With alternating microstructures on the articular membrane surface, alternating mechanical properties in the spider membrane were hypothesized to be the secret of a controlled folding of the femur‐patella joint. Using nanoindentation, a significant difference in stiffness and hardness of the two microstructure defined areas (dot and line) were experimentally proven. Due to the transparency, small size (≈3 mm^2^) and thinness (≈50 µm) of the membrane, the sample preparation and measurement was another hurdle addressed in this study. As any adhesive would result in wrinkling of the membrane or inadvertent drowning of the membrane into the adhesive, the membrane was directly attached to the aluminum holder through its thin film adhesion property. The transparency and thinness of the sample challenged the automatic surface finding and approach tool of the nanoindentation instrument, resulting in long approaching run times per indentation to evade high rate of surface detection failure. To avoid substrate effects, meaning aluminum properties mistakenly influencing the measurement results, only 1 µm of indentation depth was defined, corresponding to 2–10% of the membrane thickness.^[^ ^29^, ^30^ ^]^ The resulted mechanical properties of the articular membrane are in the same range (1–7 GPa in stiffness and 0.1–0.4 GPa in hardness) as other thin membrane measurements of arthropods (e.g., dragonfly wings^[^ ^31^ ^]^). Therefore, substrate effects can be excluded with good conscience.

Stiffness and hardness values were measured in the direction of the pressure acting on the surface when inflated,^[^ ^10^ ^]^ and potentially revealed reinforcement adaptations of the thin membrane against high internal pressure above 50 kPa.^[^ ^10^, ^13^ ^]^ To get a feeling for this pressure value, humans show a blood pressure of 120/80 mmHg corresponding to 16 and 11 kPa. Due to the size, tensile strength experiments of the different parts were not carried out but stretching and creasing of the whole dissected membrane was demonstrated (Movie S1, Supporting Information). Differences in the mechanical properties can be traced to variations in thickness between the dot and line areas as suggested by the histological longitudinal cuts. Further possible explanations may be sclerotization (cross‐linkage of proteins) resulting in stiffer material properties,^[^ ^19^, ^21^ ^]^ different embedded proteins (e.g., resilin), chitin fiber orientation,^[^ ^22^ ^]^ and microstructure effects.

Our experimental findings have confirmed the assumption of the previous researchers^[^ ^7^, ^10^, ^21^ ^]^ that the thin membrane has harder structures, i.e., modulus anisotropy. Such anisotropy can have different functions. For example, it can increase fracture toughness. Although we were unable to prove this quantitatively due to the small size of the membrane, we looked at the fiber structures and orientations (Figure 2;and Figure S2 and Movie S1, Supporting Information). The reinforced elements seem to have a thicker epicuticle, which could interrupt a tearing process (Figure 3C).

In addition, a modulus anisotropy can also be helpful to prevent or limit expansion. The inflation of pockets generally finds its optimum in spherical shapes, where inner forces created by pressure are acting on the surface equally in each direction. However, the resulting forces do not necessarily match the forces required for rotational movement, creating counter‐acting movements. This effect can be observed in some of the spider‐inspired implementations,^[^ ^7^, ^16^ ^]^ where the balloon‐like inflations reduces the working angle and torque as the spherical expansion counter‐acts the rotary movement.

Moreover, reinforced elements form a “skeleton,” which keep the thin membrane in shape and help to withstand high forces acting on the surface as it can be observed, e.g., in the sail of yaws. The stiffer elements further guide the motion along a given path like in a stroller umbrella, which could increase durability of the material due to stress reduction and decrease control complexity as the actuator behaves in a predictable way.

Several underlying principles can be extracted from our experimental observations. First, the joint actuation does not base on the balloon‐like inflation of a hyperelastic soft material as silicone, nor is it shaped as a bellow. The observed actuation bases on the folding of a fabric‐like material, which defines the maximal form, comparable to the inflation of a crumbled paper or plastic bag. Additionally, the thin membrane is equipped with hoop‐shaped stiff elements to withstand high tension during fast motions at high pressures and guides the folding of the joint membrane. This creates a controlled sliding motion, which could be beneficial for material durability and the transfer of energy for fast motion.

To test the proposed principle, a lightweight, compliant, durable, scalable, modular, and environment friendly rotary semifluidic actuator (SFA) was designed and fabricated by 3D printing. We presented a finger‐sized prototype which resembles the folding mechanism and working angle of spiders to test the biological hypothesis in an engineering context and evaluate the advantages for application possibilities in soft and flexible robotics.

The material choice needs to fulfil three requirements. First, the membrane should be as thin as possible for the folding principle of spider‐inspired design but is limited by the 3D printing parameters and material properties. Next, the material needs to be flexible. Finally, it should have low friction and stickiness to achieve the folding motion. Silicone is therefore not desirable as it likes to stick at itself and normally needs to be fabricated with a specific thickness to prevent rupture.

The lowest 3D‐printed membrane thickness is limited to 0.45 mm by the printing and slicing parameters. This means, to achieve the same bending motion as jumping spiders with a membrane thickness of 0.05 mm and a stiffness of roughly 5 GPa, a 3D‐printable material with a stiffness 1000 times lower than the stiffness of the articular membrane of spiders would be needed to create similar bending behavior as observed for spiders.

The chosen TPU filament shows a Shore Hardness of 94A, which corresponds to a Young's modulus of ≈4.8 MPa, lying in the required range. It is highly flexible and could be 3D FDM printed, although direct instead of Bowden extrusion of the material should be used.

For comparison, to achieve the same displacement when bending two beams, one with the stiffness of spider membrane and one with the stiffness of TPU, the TPU beam has to be 5–10 times thicker than the spider membrane (see the Supporting Information). This means, to fabricate a membrane similar in size of jumping spiders, a 250–500 µm thick TPU membrane would be needed to be comparable to the bending behavior of a 50 µm thick spider membrane, making the material and structural composition of spider joint membranes (chitin microfibers) an interesting subject for future research.

The 3D printed actuator weighs 6 g, has a 3 mL fluid chamber and a pivot point connection supporting working angles of 125°. In comparison to spider‐inspired models from previous literature,^[^ ^7^, ^17^ ^]^ our design shows a 30°–80° larger working angle, with a smaller fluidic chamber and less excessive material. This was only possible due to the unique fold and slide principle of the jumping spiders, as the working angle does not depend on the hyperelastic extension of the material under pressure but can be exactly predefined by the design. Furthermore, the volume of the fluid chamber is limited to only a fraction of the joint volume, reducing the weight, fastening the volume shifts, and therefore enabling fast motions (≈20 rev s^−1^ angular speed). With a variation of 15–140 mm^2^ in cross‐sectional area of the fluid chamber, the developed prototype can achieve forces up to 4–20 N, corresponding to maximal torques of 0.1–0.5 Nm at pressures of 50–300 kPa. Existing spider‐inspired fluidic actuators need high operating pressures of up to 1 MPa or 10–100 times larger cross sectional area to achieve similar torques.^[^ ^7^, ^17^ ^]^ The chosen material and the reinforcement hoops of our prototype allowed to accelerate a first multilegged testing platform for repeatable jumping motion, while keeping the whole system light and flexible, avoiding damages during landing.

The reinforcement hoops were further shown to increase durability of the actuator by guiding the folding movement and avoiding sharp and undesired wrinkles (Movie S2, Supporting Information), which could lead to material failure. Additionally, the hoops prevent the chamber from uncontrolled bulging, which could also cause leakage. In combination with the hydraulic extension, the spider‐inspired mechanism allows to keep the actuator in stable positions, while still being compliant. This enables the actuator to lift weights over 600 g, without any “wobbling” movement, that can be often observed in soft fluidic actuators due to the compressibility of air and the used “blown‐up” silicone material. Different to soft actuators, which are often pointed out to have the disadvantage of nontrivial control requirements,^[^ ^6^, ^7^ ^]^ the angle position of our prototype is only depending on the flexion wire and can therefore be simply calculated from the shortening of this wire and the material forces needed for folding. The load‐pressure behavior of our prototype at a specific angle could be approached by simple linear relationships (Figure 6A), which means that the flexion torque to keep the joint at the chosen angle increases linearly.

The joint was designed in a modular way, so that it can be easily extended for future multijoint studies. The inner latex sealing supports high pressures up to 300 kPa without influencing the performance and can be easily renewed and “healed” in case of leakage as liquid latex can be cured on itself.

Printing in one step without support allows a simpler downscaling of the prototype, as no assembly of fragile, thin, or small parts (e.g., hinge pins or shell elements) are used and weak points due to gluing or attaching components of movable parts were reduced. Material properties, 3D printing errors and the current tube connection may be limitations.

The fabrication material is completely based on environment friendly material, which becomes an important aspect when thinking about future applications.

By implementing the folding mechanism with the reinforcement into a robotic leg prototype we were able to get a better understanding of the semihydraulic actuation of spiders. In general, we showed that the combination of flexible material with stiff elements helps to keep the design in shape and guide the motion. This reduces stress peaks created by undesired sharp wrinkles which increases durability of the material and therefore avoids the cause of damage and fatal leakage in case of fluidic actuation. The guidance of motion further increases the predictability of the actuator behavior, which finally reduces the control complexity. This concept is essential to understand when designing soft or flexible elements for robotic devices especially for fast motion.

Although the flexion wire position is different to the muscle constellation in spiders, it is worth mentioning that jumping spiders keep their legs at similar positions during walking and resting.^[^ ^32^ ^]^ Full opening is observed during running and jumping^[^ ^33^ ^]^ and communication when front arms are lifted vertically, while flexing above 90° is observed during prey catching as well as jumping initialization (Movie S1, Supporting Information). In our measurements, flexion forces increase drastically when flexed above 90°, supporting the assumption that elastic deformation of the membrane might support the pressure‐induced jumping in spiders.^[^ ^34^ ^]^ In spiders, the chamber is formed by the articular membrane as well as flexion muscles connecting to and pulling at the arcuate sclerite (Figure 2). Further studies by varying the position of the flexion wire may give a better understanding of the flexion muscle attachment position, the role of the arcuate sclerite and whether a similar force increase could be observed. Furthermore, higher robot jumping performances might be achieved in the future when assembling a similar leg constellation as in spiders, allowing flexion angles above 90°.

The simulated jumping studies suggest that the jumping behavior can be adjusted by pressure, allowing the control of jumping height and flight curve by pressure and flexion angle. Considering the joint as a spring, it can be said that the spring constant changes with pressure as more energy can be stored for rotational motion with pressure increase. Different to a standard rotational steel spring or the elastic protein resilin used for jumping in insects, the semihydraulic joint allows a higher versatility in jumping performance. It can be assumed that spiders can adjust the elastic energy stored inside their jumping legs and—combined with the safety dragline—achieve a very precise jump on their prey, while the elastic energy of jumping insects is always the same at a specific flexion angle and lead to uncontrolled, catapult‐like escape motions.

During the sudden release, corresponding to muscle relaxation proposed for spider jumps,^[^ ^32^ ^]^ the fast extension of the rotary actuator creates pressure below zero, which can be explained by the inertia and viscosity of water. This pressure drop could limit the hydraulic performance and in the worst case cause cavitation, damaging the material.^[^ ^35^ ^]^ As similar effects could be expected in the spider leg during jumps, one could hypothesize that the physical properties of the hemolymph (internal body fluid) may show shear‐thinning non‐Newtonian characteristics (as the blood of humans^[^ ^36^ ^]^), where viscosity lowers with increased pressure, allowing faster fluid transfer and preventing a sudden pressure drop. So far pressure‐dependent viscosity properties of spider hemolymph have not been studied yet. As the viscosity of the hydraulic fluid has a great influence on hydraulic systems, further studies on the hemolymph properties of spiders are needed to better understand their kinematics and dynamics. A first step could be testing fluids of different viscosity and shear behavior to see whether this effect can still be observed and how the fluid dynamics influence the locomotion performance of spiders.

We further demonstrated that the proposed “inverted marionette”^[^ ^9^ ^]^ principle for spider locomotion, where muscles constantly work against the internal body pressure without could be used for jumping actuation, even without valves. The small volume chamber of the prototype allows the integration of tubes for an extension into a multijoint system. In spider joints, several channels are running down the leg to further joints,^[^ ^14^ ^]^ allowing multijoint locomotion and providing nutrients to nerves and muscles. Further experiments are needed to understand how the body fluid traverses through the leg, flows in and out the chamber and whether a proposed valve system^[^ ^10^, ^14^ ^]^ to actuate multiple joints actually exists.

The jumping spider leg joint mechanism and materials inspired us to develop a flexible semifluidic actuator and jumping robot platform. Due to its lightweight and flexible design, such actuator could be used in many robotic systems interacting with humans physically. The “hoop design,” in combination with the counteracting of fluid‐driven extension and tendon‐driven flexion, allows several stable positions, durability due to controlled folding, large working angles, and high speeds. In combination with its finger‐like structure, the prototype can be advantageous for the design of soft hand prostheses, with stable finger‐angle positioning, pressure‐dependent force generation, and fast motion as needed for many physical tasks. For future robotic applications, a flexion mechanism and a pump to create enough flexion and extension forces as well as a control mechanism for their counter play have to be integrated. Untethered small‐scale soft actuation^[^ ^37^, ^38^, ^39^ ^]^ and continuous, repeatable motion^[^ ^40^, ^41^, ^42^ ^]^ will be necessary to create fully functional robots for potential future applications in agriculture, search and rescue, environmental monitoring, and inspection tasks.

Our presented 3D actuator, leg model and prototype also provide a platform to study further spider‐related questions including the functioning and control of a multijoint and multilegged system as well as effects of fiber orientation, surface roughness, multimaterial, fluid viscosity, muscle position, and length scale.

## Experimental Section

##### Spider Keeping Condition

Juvenile jumping spiders *Phidippus regius* were purchased from a local breeder and kept in individual boxes at temperatures between 25 and 35 °C with a 9 h daylight cycle. The spiders were fed with fruit flies (*Drosophila* sp.), mealworms and crickets matching their stage in development and size. Adult spiders were used for experiments (Figure S1, Supporting Information).

##### Stereo Microscopy

Spiders were narcotized with carbon dioxide and carefully attached on a Petri dish using Dental polymer (Flexitime, Correct Flow), allowing detaching after experiments without removing many sensory hairs from the spider body. The leg segments were flexed and extended manually with a thin preparation needle and the folding of the articular membrane was observed and recorded under a stereomicroscope (Leica M205).

##### High‐Speed Video Recording

Adult jumping spiders were recorded at 1000 frames per second (Phantom Miro M140, 8032), with exposure time at 400 µs and resolution of 1024 × 768. Movie S1 (Supporting Information) shows the spider in original speed, while the jump is slowed down to 5 fps and the safety wire attachment to 10 fps, to seem more details.

##### Scanning Electron Microscopy

Traditional fixation methods for SEM such as dehydration using ethanol series and crosslinking using glutaraldehyde, do not consider that the natural joint flexes after the spider deceased. Muscle flexion results in body fluid (hemolymph) moving back into the body and the articular membrane deflates, resulting in an unnatural, partially deflated construct when reopening the joint after extension. Preattaching the whole spider also did not work out properly, as the joints still curl and deflate, and tested adhesives dissolve during the Ethanol or Acetone washing steps. Therefore, we developed a method for this procedure. The spider was sedated (not killed) using carbon dioxide (Figure S1, Supporting Information) for 10 min and directly transferred into a solution of 70% water, 20% ethanol, and 10% ethyl acetate for 10 min. Ethyl acetate vapor is a common killing agent of entomologist and known to resoften joints of dead insects for collection preparation. When taken out from the solution, the spider was completely soft and flexible. A small Petri dish (2 cm diameter) was filled with water and the spider was transferred carefully on the water surface. Surface tension of water and the slightly hydrophobic characteristic of the solution fully stretch the spider on the water surface, separating the legs from each other. The spider was then lifted out from the water using a thin metal plate (Figure S1, Supporting Information). For jumping spiders with large abdomen and short legs, the legs were lifted into a horizontal position using hot glue below as a base and for attachment. By pulling small hot glue strings around the tarsus, forming thin hot glue handcuffs, the legs were mechanically locked, when the hot glue loses its stickiness in ethanol during the critical point drying (1 h, slow speed, 28° on Leica EM CPD300 Automated Critical Point Dryer). In a last step, the spider was sputter‐coated with 20 nm gold (Leica EM ACE200) to avoid charging effects under the electron microscope (Zeiss Ultra Plus) with working distances of 10–15 mm and electron high tension of 3–5 kV.

##### Histology

Hartman's fixative (also Davidson's fixative, Sigma‐Aldrich H0290) often used in vertebrate tissue fixation, was used for sample fixation. The fixative contains acetic acid, alcohol, and formalin. Samples were fixated overnight. To avoid rupture of the articular membrane, paraffin embedding was chosen. Further fixation and embedding methods were tested but examined as not suitable for spider leg histology (see the Supporting Information). The following protocol was optimized for thin and soft samples with large variances in material properties to allow successful histological cuts.

Paraffin wax was heated in a beaker and filled into small clear lockable glass sample vials and left on the heating plate. Samples were incubated in a glass vial filled with xylene (also xylol, Sigma‐Aldrich, isometric mixture, 1.08 298) for 10 min. Longer incubation could lead to brittleness of the sample. Samples were transferred into a 1:1 xylol‐paraffin wax mixture and incubated for 10 min. Afterward, the samples were incubated in fresh paraffin for 30 min twice. For cutting purposes, samples were transferred into stainless steel histology embedding molds, covered with the backside of a plastic histology cassette and filled with liquid paraffin. After cooling in air (1–2 min), molds were cooled in the fridge (−20 °C) overnight. Slices of 4–5 with 4 µm thickness were cut with the microtome (RM2245, Leica) and transferred (Figure S1C, Supporting Information) with a brush to a heated water bath (35–40 °C) and carefully lifted on a glass slide to be baked at 45–50 °C overnight. For staining, paraffin wax was removed by bathing the slides in xylene (10 min), hydrated with ethanol washing (96%, 70%, 60%, 5–10 min per step) and rinsed with distilled water afterward. Three different staining methods were tried out, Mallory (Morphisto, Mallory Kit, 10270), Masson‐Goldner and Azan staining. Best staining results were obtained with a modified Masson‐Goldner Trichrome staining. Staining jars were used for all staining steps. Weigert's iron hematoxylin solution from the traditional Masson‐Goldner staining was not used as it is for nuclei staining. Samples were bathed in a 1:2 mixture of 1% acid fuchsine (Morphisto, Masson A, 10357) and 1% Ponceau de Xylidine (Morphisto, Masson B, 11518) for 5 min and carefully washed with 1% acetic acid and distilled water afterward. Samples were then shortly (1–2 min) dipped in a solution of phosphormolybdic acid and Orange‐G (Morphisto, 11 195) and washed with acetic acid and distilled water again. After another staining step with anilin blue (Morphisto, Masson C, 10 141) and washing, samples were quickly dehydrated with ethanol (96%, 5 min, twice) and xylene (2 min) and left under the fume hood for drying and total evaporation of xylene. Cover slides for microscopy were mounted with mounting medium (Morphisto, 12 318) onto the glass slides. Coloration results show similarities to Azan staining after Heidenhain. The thin epicuticle, which is not stained in many other staining protocols, is stained dark red with this method. The stiff exocuticle appears brown, while the endocuticle is stained blue. A mesocuticle, which does not show fiber orientation differences to the endocuticle but can be differently stained,^[^ ^22^ ^]^ was not distinguishable with this method.

##### Deproteinization Quick Test

To analyze the base material of the articular membrane and its microstructures, proteins have been removed from the exoskeleton. Therefore, spiders were deproteinized by boiling in a 10% KOH solution (modified according to Barth^[^ ^22^ ^]^). Faster, microwave‐assisted deproteinization methods have been proposed recently,^[^ ^43^ ^]^ but would have been too aggressive for articular membrane studies. To prepare the solution, ultrapurified water was boiled, and KOH drops were added under stirring. Boiling chips were added to avoid large bubble formation. A watch glass was used to minimize evaporation. Samples were boiled for 2–4 h depending on sample size and rinsed with ethanol afterward. When adding a whole spider into the solution, the spider inflates and air escapes out of the abdomen, propelling it along the water surface. After the boiling process, all hair and colors were removed, leaving a transparent, chitin fiber‐like construct of the exoskeleton behind (Figure S2, Supporting Information).

##### Indirect Resilin Quick Test

In a second quick test, a possible existence of resilin in the membrane was evaluated. This indirect test uses the autofluorescence property of resilin under UV light (385nm).^[^ ^25^ ^]^ Samples were observed under a stereomicroscope (Leica, M205 FA) with fluorescent light source (EL600, mercury lamp) and filter (EX 395/25 nm, EM 435 LP). Additionally, higher magnified images on an inverse microscope (Zeiss Axio Obser.A1) with UV light (385 nm) and filter (Ex 390/40, Em 450/40) were recorded. While strong blue fluorescence indicates resilin existence, the observed light blue color could also be attributed to nitrogen‐rich cross‐links between the epicuticle.^[^ ^24^ ^]^ Future studies, as observing the pH‐dependence of the autofluorescence, could help to verify the existence of resilin.^[^ ^44^ ^]^

##### Nanoindentation Tests

Using carbon dioxide, spiders were narcotized and fixed with straight legs on a Petri dish using Dental polymer, Vinylpolysiloxan (Flexitime, Correct Flow). The legs were carefully cut off and the thin articular membrane between the femur‐patella joint was dissected under the Stereomicroscope (Leica M205. The membrane was placed on a 3 cm diameter aluminum block. As the membrane is very thin and deformable, a water droplet was used to adhere the membrane on the surface. Other adhesives were tested but the curing methods resulted in nonflat samples, or samples drowned in adhesives.

The mechanical properties of the articular membrane were measured via nanoindentation (Nanoindenter XP, Keysight) with a pyramidal Berkovich tip (JDA, 1 µm) (Figure S1, Supporting Information). The hardness and the Young's modulus of the two distinguishable parts (dot and line area) of the membrane were determined by the continuous stiffness measurement (CSM) method. As the indenter had only a fixed 20x magnification, it was difficult to find the small and transparent membrane under the microscope. Indentation points were chosen randomly among the surfaces but avoiding wrinkles. For noise balancing and calibration, 5 indents on a silicon wafer have been taken and for each area of the membrane 5–10 test indents have been performed to mark the xy‐position of the different parts. An indentation depth of 1 µm was chosen, corresponding to 2–10% of the membrane thickness avoiding substrate effects.^[^ ^29^ ^]^ Further parameters for the nanoindentation experiment are summarized in Table S2 (Supporting Information). Two experimental runs were performed. In the first run 50 and in the second 100 indentation runs for each area were done. Only experiments in accordance with theory,^[^ ^30^, ^45^, ^46^ ^]^ have been chosen for calculation, to ensure successful contact finding and avoiding substrate effect. At the end, 57 measurements for the dot and 101 for black line area were analyzed. Statistical analyses (Table S1, Supporting Information) were performed using R internal statistic libraries.

##### Design and Prototyping

Different methods and materials for fabrication were tested (Table S4, Supporting Information) including 3D printing. 3D modeling was performed using the CAD software Autodesk Fusion 360. The models were sliced using the Ultimaker software CURA for the 3D FDM printer (Creality Ender‐3 Pro). Printing parameters can be found in Table S3 (Supporting Information). The chosen TPU filament (EoFlex by EOLAS, Reference 1 009 600 000) shows a tensile strength of 40 MPa and has a shore hardness of 94A. To improve performance for flexible material prints, the bowden extruder was moved directly on top of the printing head (Figure S1, Supporting Information). For flexion, 0.12 mm thick fishing yarn (Spiderwire, Stealth Smooth 8) was used. For further sealing 3D print irregularities, different materials have been tested (Table S5, Supporting Information). Latex milk (Laguna, natural liquid latex rubber, low ammonia (<0.3%), 60% solid content) was determined to be most successful for sealing purposes. By filling the chamber with Latex milk and curing it for 30 min at 50° in the oven, a thin layer covers the 3D print on the inside, filling up the printing irregularities. If cracking occurs, sealing can be renewed by drying the leg and refilling latex milk into the leg as latex milk “partially dissolves” older latex surfaces and cures on itself.

##### Prototype Characterization

For the folding behavior and durability test, the flexion wire was attached to a 12 V‐stepper motor (NEMA 17) and flexion and extension runs (2 × 500 cycles) for each leg were performed (run time: 2 s per cycle). For flexion characterization, an experimental set‐up was 3D printed (PLA), including a load sensor platform and a rotational flexion chamber. A force sensor (5 kg load cell with HX711 AD amplifier) and pressure sensor (30 PSI, ≈200 kPa, stainless steel, GS01525, DC‐5V) attached to an Arduino Uno (Figure S1, Supporting Information) were used to measure load and pressure. Before every measurement, the force and pressure sensor were calibrated.

The flexion mechanism consisted of a water reservoir with a Latex inner bladder, which fills up when the joins fold. During flexion, the wire rolls around the cylinder. By releasing the wire, the bladder is pushed by an inner cylinder, so that the water fills the joint. As flexion wire, a fishing yarn (Spiderwire, Stealth Smooth 8) was used to avoid curling. Tracking markers were attached to the leg and the base (fixation) and the leg movement was recorded. The tracking angle was analyzed using the software Tracker (physlets.org/tracker/). The video and the sensor measurement were synchronized by hitting the force sensor with a pen to achieve a visual signal that could be correlated to a spike in the force sensors. Legs were flexed and released slowly (6 experimental runs) and suddenly (4 experimental runs) by detaching the wire from the flexion mechanism.

For characterization of the extension property a hinge platform (Figure S3, Supporting Information) was 3D printed (PLA). Legs were hold in the stable positions (40°, 75°, 100°, 125°) and pressure was increased with a syringe up to 200–300 kPa. Load was recorded. For each position, 3 experimental runs were conducted. At position 75°, tube leakage occurred during one experimental run and was therefore excluded from analyses. Position 100° was tested 5 times in total as it was considered as the critical position for jumping and lifting experiments. All sensors (pressure sensor: 100 PSI, ≈690kPa; load cell: 5 kg, ≈50 N) were attached to an Arduino Uno. Leakages through tube connections were minimized by shorter PVC tubes and push‐in tube connectors. As commonly done for hydraulic systems, pressure sensor attachment sealing was improved by roughening the tube connector thread and covering it with a polytetrafluoroethylene (PTFE) tape.

##### Statistical Analysis

Statistical methods were used to analyze the results of the nanoindentation experiments (Table S1, Supporting Information): *t*‐test, was used to test whether the mean of two normal distributed groups with the same variance are equal. Shapiro‐test verifies the normal distribution, and F‐test compares variances of two sample groups, assuming normal distribution. Welch test is a modified *t*‐test for samples showing different variances. Mann–Whitney–Wilcoxon test was used for testing the identity of two distribution without assuming a normal distribution to make a statement about the independence of two samples.

## Conflict of Interest

The authors declare no conflict of interest.

## Author Contributions

C.G. and M.S. proposed and designed the research; C.G. carried out all designs, prototyping, experiments, data analysis, and manuscript writing. K.E. optimized procedures for the histological dissection of the leg. R.S. and M.S. supervised the research. M.S. contributed to the writing of the manuscript. All authors have given their consent to the final version of the manuscript.

## Supporting information

Supporting Information

Supplemental Movie 1

Supplemental Movie 2

Supplemental Movie 3
