# Supplementary material for: Spider Origami: Folding Principle of Jumping Spider Leg Joints for Bioinspired Fluidic Actuators
Source: Adv Sci (Weinh). 2021 Jan 21;8(5):2003890. doi: 10.1002/advs.202003890 (PMC7927609; doi:10.1002/advs.202003890)
Supplement: Supplementary file 1 — Supporting Information [file ADVS-8-2003890-s001.pdf]

© 2021 Wiley-VCH GmbH

ADVANCED SCIENCE
Open Access

Supporting Information

for *Adv. Sci.,* DOI: 10.1002/advs.202003890

Spider Origami: Folding Principle of Jumping Spider Leg Joints for Bioinspired Fluidic Actuators

*Chantal Göttler, Karin Elflein, Roland Siegwart and Metin Sitti**

Supplementary Information

for

Spider Origami: Folding Principle of Jumping Spider Leg Joints for Bioinspired Fluidic Actuators

*Chantal Göttler, Karin Elflein, Roland Siegwart and Metin Sitti**

* E-mail: sitti@is.mpg.de

The Supplementary Information includes:

Text
Fig. S1. Material and methods overview.
Fig. S2. Potassium hydroxide-treated spider leg samples.
Fig. S3. Stable positions of the actuator prototype.
Table S1. Statistical analysis of the nanoindentation measurements.
Table S2. Nanoindentation test parameters.
Table S3. 3D printing parameters.
Table S4. Tested materials and fabrication methods.
Table S5. Tested sealing materials and methods.
Table S6. Linear fit for load-pressure behavior estimation

Other Supplementary Material for this manuscript includes the following:

Movie S1 (.mp4 format). Articular membrane characterization
Movie S2 (.mp4 format). Prototype characterization
Movie S3 (.mp4 format). Demonstration of lifting and jumping performance

Supplementary Text

S1. Biological details of arthropods

The group (taxon) of *Arthropods* evolved around 550 million years ago and includes invertebrates with an outer shell, the exoskeleton, as insects, crustaceans and arachnids, covering around 80% of all known living animals. The fundamental material of the exoskeleton is chitin, a polysaccharide similar to cellulose. Layers of chitin microfibers are held together by different proteins to form the exoskeleton and depending on the number and arrangement (hierarchical structure) of these layers and the type of proteins, the material properties of the exoskeleton can vary from flexible and soft as in caterpillars to stiff and strong as in beetles. The exoskeleton (cuticle) of arthropods can be divided into three distinct parts: the epi-, exo-, and endocuticle. The exocuticle is often highly sclerotized (stiffened) and can be followed by an intermediate between exo- and endocuticle, the mesocuticle. The endocuticle forms the innermost part of the exoskeleton, containing non-sclerotized soft microfiber-chitin-protein-layers. It plays an important role in the growth of arthropods during the formation of a new exoskeleton for molding.

As different from vertebrates, arthropods have an open circulatory system. In a closed-circulatory system as in humans, the heart pumps the blood along defined capillaries supplying the body and its organs with oxygen. Interstitial fluid filling the space between blood vessels and cells forming their own circulation, the so-called lymphatic system, exchanging and providing nutrients such as sugar and salt, hormones and enzymes. In arthropods, there is no distinction between blood and interstitial fluid. The body fluid (hemolymph) fills up all cavities (hemocoel) inside the body and is pumped by the heart through the body.[1]

Spiders (Araneae) form the largest group among the class of arachnids, which also includes other eight-legged invertebrates, such as harvestmen, scorpions and mites. Spiders consists of two body parts, the prosoma and abdomen, which are linked together by a thin connection tube (petioles). The abdomen, also called opisthosoma, is a soft inflated bag containing the heart. The front body part (prosoma) is formed by two shells, the lower shell (sternum) and the upper shell (carapax). The eight legs are connected in between these two shells. Each leg is divided into seven segments (Figure 1B) and contains around 30 different muscles.[2] The leg can be broken down into three main joints.[3] Two of these main joints (femur-patella, tibia-metatarsus), crucially involved in grasping and jumping, are lacking extensor muscles. Parry and Brown showed that the pressure inside the legs of spiders increases from 6 kPa to 60 kPa in all legs simultaneously when the spider starts moving.[4, 5] They concluded that the increase in pressure is the main driving force behind the extension of the spider leg, capable of producing torques up to 0.013 Nmm at 50 kPa.[4] Several researchers proposed and experimentally showed that this pressure is not generated by the heart but in the prosoma, presumably by muscles moving the two shells towards each other creating a volume shift.[6–9] The joint structure of the spider legs plays a key role in the adaption of the hydraulic principle into a robotic application. The working principle of the spider tibia-metatarsus joint has been described by Blickhan and Barth as a bellow-like folded, anisotropic articular membrane which stores energy when folded and inflates with the increase in pressure. Thereby, the anisotropy of the membrane avoids opposing torques by reducing the axial stress components in the joint membrane as in an isotropic membrane, pressure would result in torques counteracting the extension direction.[10]

S2. Materials and Methods

S.2.1 Histology

Several fixation, embedding and staining techniques have been tested. Spiders were narcotized with carbon dioxide and attached to a petri dish with dental polymer. Legs were

quickly cut off from the body and a targeted stitch through the abdomen (heart and digestive organs) killed the spider. Freeze killing or ethyl acetate as killing agent influenced the preparation results and could not be used. A petri dish with wet tissue (Ringer's solution) was prepared and the femur-patella joint was carefully stretched and hold in positions with insect needles inside. To keep this position during sample fixation and embedding, a thin insect needle was stitched through the leg segments near the hinge joint side (Fig. S1). Traditional dehydration with ethanol (30, 60, 70, 90, 96, 99%) was tried with variation in incubation time (5, 10, 30 min per step), but all fixation trials resulted in too brittle and stiff samples. Similar results were observed when fixated with glutaraldehyde and osmium. A fixation with picric acid (Bouin solution[11]) resulted in bright yellow samples (Fig. S1C). Although this method was great for microscopic observations, the picric acid has to be washed out with ethanol after staining. Therefore, to spare the samples, Hartman's fixative (also Davidson's fixative, Sigma-Aldrich H0290) often used in vertebrate tissue fixation, was used. The fixative contains acetic acid, alcohol and formalin. Samples were fixated overnight. Embedding in epoxy (Technovit) and spurr were also tested, but microtome cutting (with (sheet) glass, razor blades and (cryo) diamond knife, 4 mm, 45°, Leica) resulted in rupture of the articular membrane due to too large cutting thickness (~2 µm), the thinness of the membrane and large differences between the stiffness of the sample and the embedding material. Paraffin embedding was therefore chosen and carried out in the standard way.

S.2.2 Jumping performance estimation

Pressure is defined as the force acting on a surface area. To estimate the load - pressure behavior of our system, a constant contact area at each stable position (Fig. S3) was assumed, resulting in a proportional increase of load with pressure. Linear fits through the experimental data at each stable position (Fig. S3) have been carried out (Table S6). Displayed linear functions (Figure 6A) show linear fits with a mean slope of 0.013 N/kPa, corresponding to a contact surface of 13 mm². This surface would match to the surface at the entry of the chamber, were water supply is connected to (Figure 4, Fig. S3). Further studies of this surface area have to be done in following works.

The mean slope value was used to calculate the load at 200 kPa (Figure 6C) and the resulting work (Figure 6D), mirrored by the sum of the area below the curve (W1, W2, W3, W4). The resulting work shows a quadratic behavior with opening distance as:

$$W = 105.86s^2 + 1.6731s\,. \tag{1}$$

As the work is the integral of force over distance, a linear force-distance behavior, would explain this quadratic function and shows that the initial flexed position s of the legs has a great impact on the jumping performance. This formula was used to estimate the jumping height, whereby the work created by the elastic material was subtracted as it is needed to just lift the platform. The elastic work for four legs lies between 0.17 Nm to 0.42 Nm for legs flexed between position 75° and 100°. For calculation purposes, the mean-value (0.30 Nm) was used. The work left after deduction was treated as potential energy of the system, to estimate the jumping height as:

$$h = \frac{\Delta W}{mg}\,. \tag{2}$$

S.2.3 Material behavior comparison

The chosen TPU filament shows a Shore Hardness of 94A, which corresponds to a Young's modulus E of approximately 0.005GPa (4.8 MPa) from

$$E = e^{(ShoreA*0.0235-0.6403)} \tag{3}$$

For comparison, to achieve the same displacement s when bending two beams, one with the measured stiffness of spider membrane (5 GPa) and one with the stiffness of TPU (5 MPa), keeping length L, width b and applied force F of the beams the same, the thickness d of the TPU beam has to be 10 times higher than the spider membrane, computed from:

$$s = \frac{-4L^3}{Ed^3bF} \; . \tag{4}$$

Assuming the measured stiffness for the articular membrane could be 10 times lower under hydrated condition, it would still result in a ratio of 5. This means, to fabricate a membrane similar in the length scale of jumping spiders, a thickness of 250 µm of TPU membrane is needed to be comparable in its bending behavior to a 50 µm thick spider membrane.

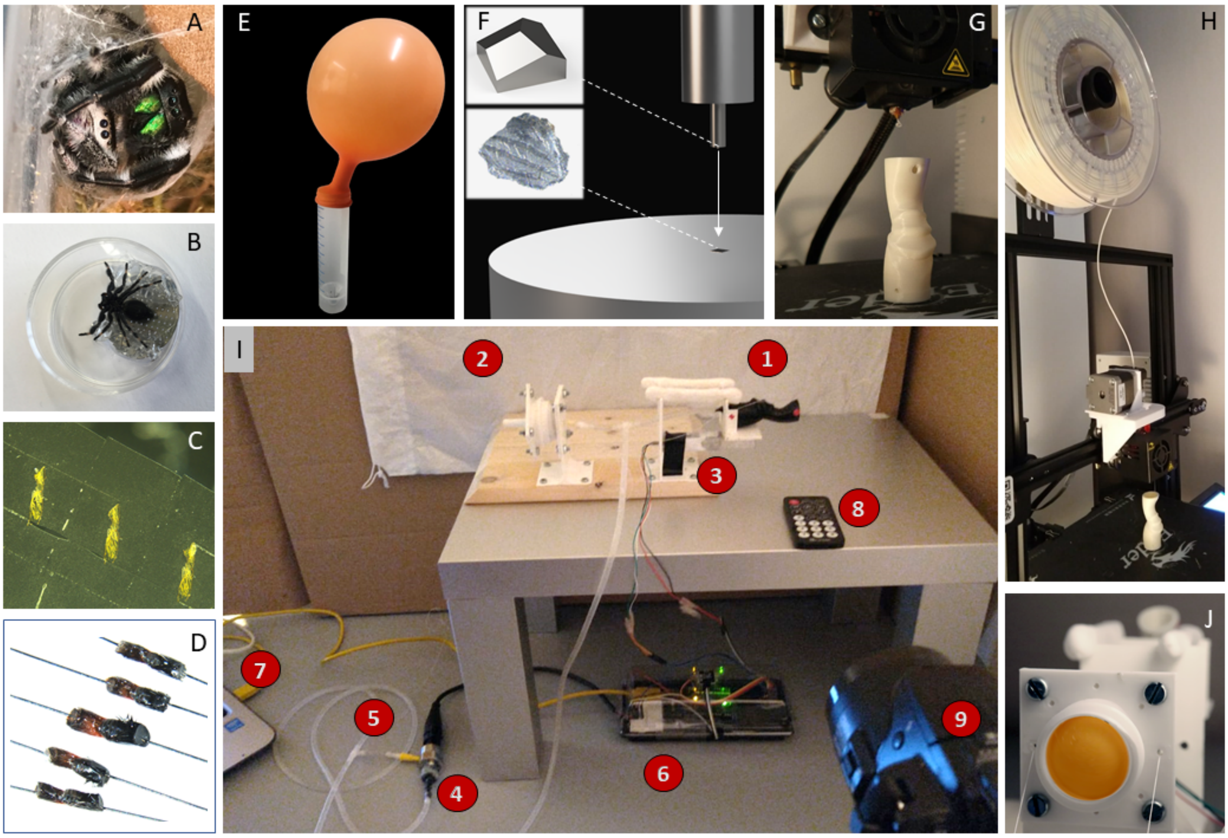

Fig. S1. Material and methods overview. Jumping spiders *Phidippus regius* were bred for experimental studies. Photo shows female (white) and male (black) adult spider after copulation (90min) in the female's "sleeping bag". Male was "chased out of the house" shortly after taking the photo. Female abdomen increased in size drastically and the mother-to-be formed a dense net inside her "sleeping" bag, where juvenile spiders developed within a couple of weeks (A). For SEM preparation the dorsal side of the spider was attached to a thin metal sheet. Hot glue was used to lift the legs into horizontal position. Small hot glue "hand cuffs" were formed around the feet to create a mechanical interlocking (B). First histological experiments were tested with a picric acid solution, resulting in bright yellow paraffin cuts, good for microscopical observation, but not for staining purposes (C). To avoid flexion of the femur-patella joint during preparation and cutting, thin insect needles were stuck through the leg segment resulting in small spider leg "resistors" or "BBQ sticks". To avoid damage on the membrane side, needles were stuck through near the hinge joint side (D). For narcotization, spiders were transferred into a falcon tube and a balloon was filled with carbon dioxide (E). Nanoindentation of articular membrane (lower left) was conducted with a Berkovich tip (upper left) on aluminum substrate (F). 3D model of spider-joint-inspired actuator prototype is printed in vertical direction (femur to patella) to avoid support material inside the chamber (G). Bowden extruder head was rebuilt to direct extrusion, allowing better 3D printing of flexible material as rubber material show discontinued material pushing due to its elasticity properties (H). Experimental set-up for prototype characterization includes, the prototype with red tracking markers (1), a rotating flexion mechanism (2), a force sensor (3), a pressure sensor (4), a connection for a syringe to increase pressure (5), an Arduino Uno for sensor processing (6), a computer for recording (7), a remote control for changing into calibration mode (8) and a camera, recording folding angle (9) (I). The flexion mechanism consists of an inner latex bladder that inflates and collects the water, when the leg is flexed (J).

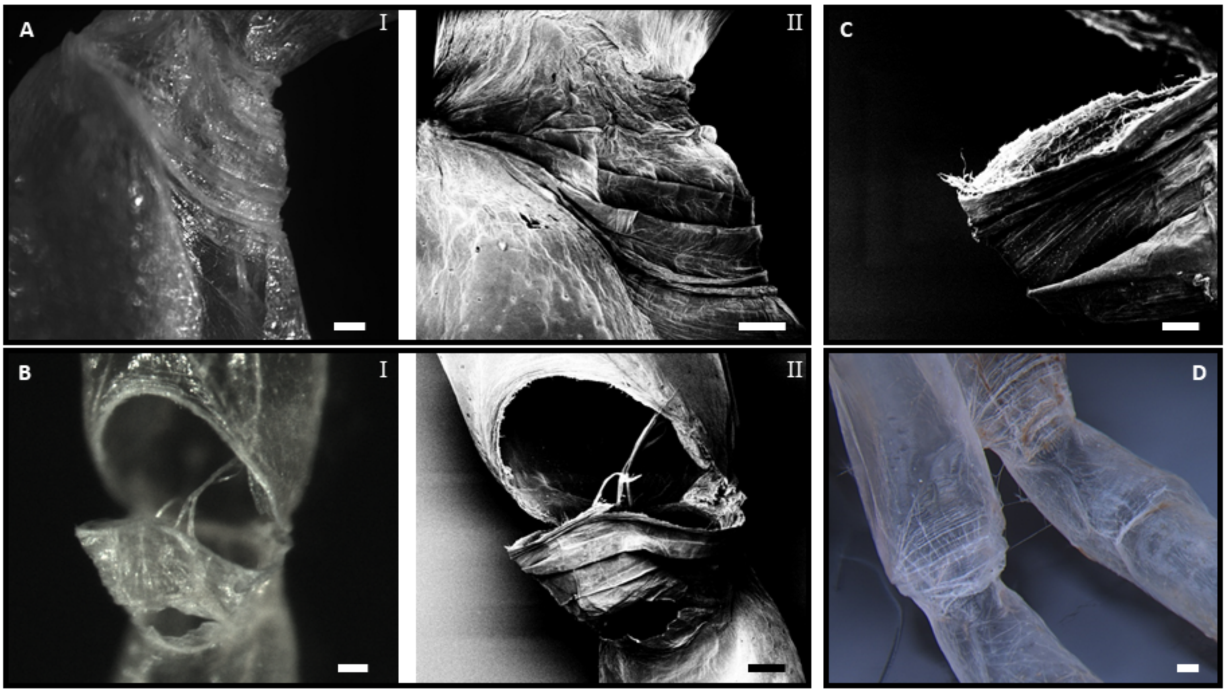

Fig. S2. Potassium hydroxide-treated spider leg samples. Spider boiled in 10%-KOH solution, leaving a transparent, stable chitin construct behind, with articular membrane inflated (D). Legs were carefully cut off for microscopy and positioned in different angles. Spider legs observed under Stereomicroscope (A-I, B-I) and SEM (A-II, B-II) after sputter coating. Images show a fabric or paper like folded chitin construct when slightly flexed (A, B). Articular membrane is thinner than the leg segment and shows chitin fibers when separated from femur-segment (B, C). Scale bar: 100 µm.

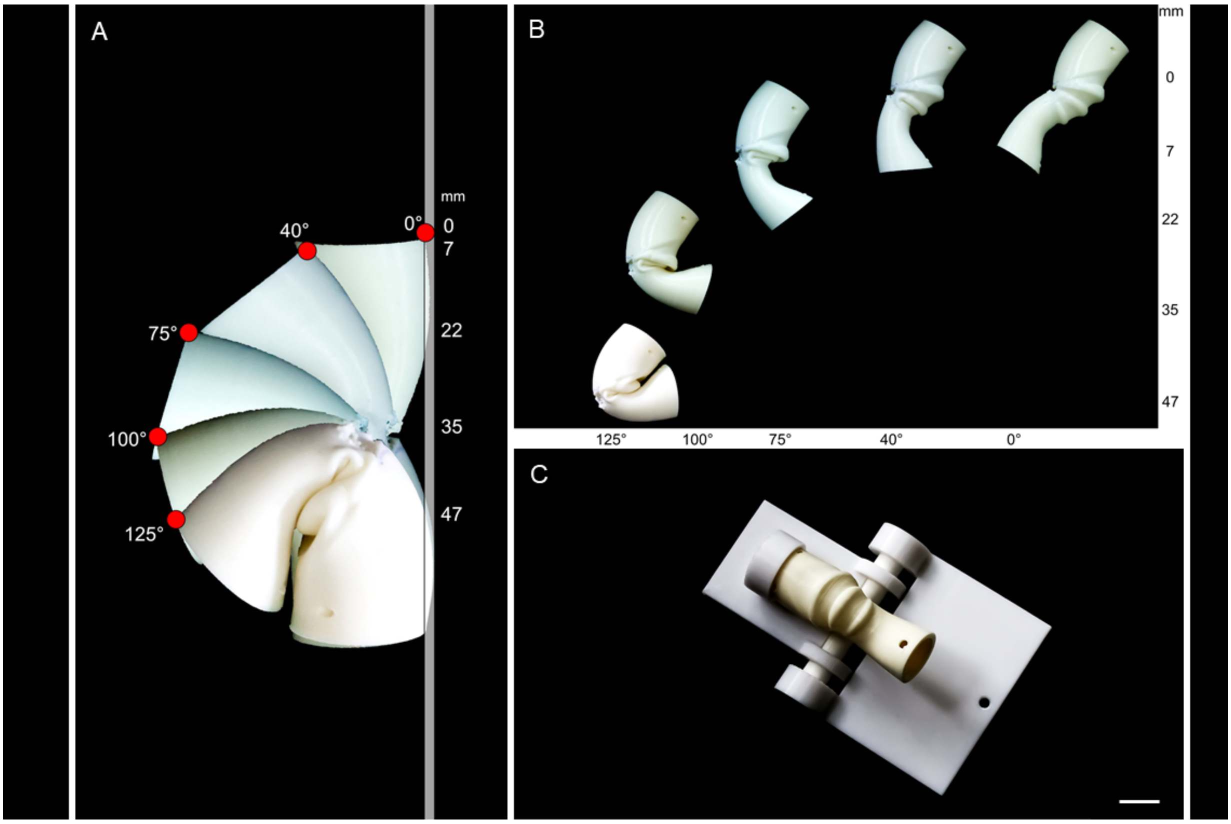

Fig. S3. Stable positions of the actuator prototype. Similar to collapsible silicone bowls, the prototype shows five stable positions (0,40,75,100,125) created by the design and elastic material. The angles correspond to opening distances, for a ("patella")-segment length of 30 mm. On the photography position 40 is not fully fold in, the shown angle is therefore closer to 35° (A). Spiders show leg angles between the shown position 40 and 100 during walking and resting. Full closing (position 125) can be observed in dead spiders, when muscles are flexed, as well as in jump initialization. Spiders communicate with completely opened (position 0) front legs, showing their articular membrane to their fellow species. Fully extended femur-patella joints can also be seen in the highspeed recordings (movie S1) of spider jumps (B). To measure the pressure-load-angle behavior at the stable positions, the prototype was clamped between the two plates to a given angle. The bigger plate is attached to a load sensor, the leg to a syringe and pressure sensor. Sensor data were recorded with an Arduino Uno, while pressure was increased with the syringe. Scale bar: 10 mm (C).

Table S1. Statistical analysis of the nanoindentation measurements. The distribution of Young's Modulus E and Hardness H experimental data were analyzed, p-values of the statistical tests are listed. Statistical testing was conducted with R. Normal distribution is tested with the Shapiro-Wilk-Test and the F-Test compares variances of two normal distributed groups. P-values above the significance level of 0.05 in these two tests are marked with an asterisk, indicating normal distribution or no difference in variance. Biological samples are often considered as normal distributed with similar variance, even when experimental data show different. As both, Shapiro- and F-Test are not clearly supporting normal distribution and similar variances, a standard t-test cannot be used to test similarity in means. Therefore, the Welch's t-Test is used, which considers differences in variances and the Wilcoxon-Test compares groups without assuming normal distribution. P-values, below the significance level 0.05 are marked with an asterisk, indicating a difference in means of the dot and line area.

Young's-Modulus E (GPa)	**Mean (GPa)**	**Median (GPa)**	**Standard Deviation (GPa)**	**Shapiro-Wilk-Test p – value**
**dot area (D)**	3.81	3.80	0.538	0.24 *
**line area (L)**	5.00	4.90	0.960	0.000114
**F-Test**	**ratio of variance**	0.31		
	**p-Value**	6.23e-06		
**Welch's t-Test**	**95% confidence interval**	[-1.42, -0.95]		
	**p-Value**	< 2.20e-16 *		
**Mann-Whitney Wilcoxon Test**	**p-Value**	6.40e-15 *		

Hardness H (GPa)	**Mean (GPa)**	**Median (GPa)**	**Standard Deviation (GPa)**	**Shapiro-Wilk-Test p – value**
**dot area (D)**	0.12	0.11	0.041	0.000446
**line area (L)**	0.29	0.29	0.045	0.22 *
**F-Test**	**ratio of variance**	0.85		
	**p-Value**	0.509 *		
**Welch's t-Test**	**95% confidence interval**	[-0.18, -0.15]		
	**p-Value**	< 2.20e-16 *		
**Mann-Whitney Wilcoxon Test**	**p-Value**	< 2.20e-16 *		

Table S2. Nanoindentation test parameters. Parameters used for Nanoindenter (XP, Keysight) and data analysis for hardness and Young's modulus. Poisson ratio was chosen according to literature.[12] Depth limit corresponds to 2-10% of sample thickness to avoid substrate effects.[13]

Parameter	Value	Unit
Surface approach velocity	2	nm/s
Surface approach distance	1	µm
Strain rate	0.05	1/s
Harmonie displacement	2	nm
Frequency	45	Hz
Depth limit	1	µm
Surface stiffness	160	N/m
Poisson ratio	0.4[12]	-
Max. Depth for Modulus Av.	150	nm
Min. Depth for Modulus Av.	100	nm
Max. Depth for Hardness Av.	600	nm
Min. Depth for Hardness Av.	500	nm

Table S3. 3D printing parameters. Parameters used for fused deposition modeling (FDM) with Creality Ender-3 Pro. 3D model designed in Autodesk Fusion 360 and stl-file directly exported to CURA (Ultimaker). The software slices the model and generates G-codes for 3D printing.

Parameter	Value	Unit
Layer Height	0.2	mm
Wall Thickness	1.0	mm
Print Thin Walls	Enabled	-
Infill Density	100	%
Infill Pattern	Concentric	-
Printing Temperature	205	°C
Build Plate Temperature	25	°C
Flow	100-150	%
Print Speed	50.0	mm/s
Retraction	Disabled	-
Fan Speed	100-120	mm/s
Build Plate Adhesion Type	Skirt	

Table S4. Tested materials and fabrication methods. Several materials and fabrication methods were tested to investigate the most promising approach for a spider-inspired 3D prototype. The table summarizes observations and limitations of tested methods.

**Material**	**Comment**
Textile	• If not water repellent, sealing with different methods as wax or glue necessary<br>• Limitation: thick material, stiffness variance by creating inlets with e.g. metal sticks as in stroller sunshades is time consuming and not scalable, high friction
PE foil	• Cheap<br>• Bags can be formed using heat or glue<br>• Good prototyping material<br>• limitation in (air) pressure and sealing
Other foils	Nylon<br>• Stitching, laser or glue processing<br>• Limitations: sealing issues<br>Foil balloons<br>• Easy processing material with heat<br>• Limitation: only one side can be heat treated
Silicon	• Not for fast prototyping due to time consuming molding/casting<br>• Pressure resistance ("blowing up") and thinness might be limited<br>• Good sealing quality<br>• Useful as comparative material for future work for given design<br>• 3D printing techniques are now evolving
Latex	• Not for fast prototyping due to time consuming molding/casting<br>• Pressure resistance ("blowing up") and thinness might be limited<br>• Good sealing quality, can be resealed<br>• Useful as comparative material for future work for given design
Carbon fiber	• Time consuming in fabrication<br>• Too thick or not flexible enough for desired purposes
Paper	• Complex 3D shapes by origami techniques<br>• Easy processing for "quick and dirty" prototype testing<br>• Limitation: Sealing, fractures forming after several usage<br>• Might be useful in combination with a good sealing material
Tango Black	• Flexible, but sticky<br>• High resolution and two material printing due to PolyJet technique possible<br>• Not completely sealed<br>• Limitation in thinness, rips easily during 3D print cleaning, non-water-soluble support material leads to time consuming washing<br>• Useful as comparative material for future work for given design
TPU	• Available for FDM 3D printing, allowing fast prototyping and complex shapes<br>• Comparatively cheap flexible material and fabrication method<br>• Limitation: 3D printing set-up not fully evolved, can differ highly from 3D printer type, 3D prints need to be fully sealed as irregularities can occur

Table S5. Tested sealing materials and methods. Several materials and fabrication methods were tested for sealing purposes. Table summarizes observations and limitations of tested methods.

Material	Comment
Silicon	Smooth-On Ecoflex<br>• Does not cure on the TPU surface
Plasti-Dip	Covering 3D print by dipping<br>• Fast fabrication, but thickness varies with viscosity limiting folding<br>• Changes TPU material, gets brittle<br>• Material not environment friendly<br>Spray<br>• Thin layer, movements lead to peeling off
Leakage Spray	Stop Leakage<br>Weicon, Universal Dicht-Spray (Allround Sealing Spray)<br>• Gray, flexible sealing layer, good adhesion on TPU<br>• Thickness varies<br>• Small 3D irregularities cannot be fixed
Latex	Laguna, natural liquid latex rubber, low ammonia (<0.3%), 60% solid content<br>Covering 3D print by dipping and drying at room temperature<br>• Sticky layer, using baby powder to avoid self-sticking and peeling off, curing times vary<br>• Difference in thickness when drying through draining<br>• Small bubbles can form on the surface when dipping, leading to unsealed area<br>• Peeling off can happen when pressurizing<br>• Can influence “collapsibility” behavior due to thickness on the outside<br>Filling chamber and baking at 50° for 30 min<br>• Non-sticky inner layer, thin sheet of latex<br>• Holes can be “healed”, by repeating the process<br>• No influence of the folding properties

Table S6. Linear fit for load-pressure behavior estimation

position (°)	fixed intercept (N)	fitted slope (N/kPa)	R-value	p-value
40	1.5	0.017	0.931	< 2.2e-16
75	3.5	0.012	0.890	< 2.2e-16
100	6.0	0.012	0.795	< 2.2e-16
125	10.0	0.012	0.905	< 2.2e-16
	**mean**	**0.01311004**		
	**fitted intercept**			
40	1.47	0.017	0.750	1.2e-15
75	4.29	0.007	0.576	2.9e-07
100	5.89	0.012	0.520	6.0e-14
125	10.57	0.009	0.627	1.6e-12

References

[1] K. Huckstorf, G. Kosok, E. ‑ A. Seyfarth, C. S. Wirkner, Zool. Anz. J. Comp. Zool. **2013**, 252, 76.

[2] R. F. Foelix, Biologie der Spinnen, G. Thieme, Stuttgart **1992**.

[3] T. Weihmann, M. Günther, R. Blickhan, J. Exp. Biol. **2012**, 215, 578.

[4] D. A. Parry, R. H. J. Brown, J. Exp. Biol. **1959**, 36, 423.

[5] J. F. Anderson, K. N. Prestwich, Z. Morph. Tiere **1975**, 81, 257.

[6] C. Kropf, Hydraulic System of Locomotion Spider Ecophysiology (Ed: W. N. entwig), Springer, Berlin Heidelberg **2013**, pp. 43–56.

[7] R. Blickhan, F. G. Barth, J. Comp. Physiol. **1985**, 157, 115.

[8] R. S. Wilson, Z. Morph. Tiere **1970**, 68, 308.

[9] J. Runge, C. S. Wirkner, Zool. J. Linn. Soc. **2019**, 186, 353.

[10] R. Blickhan, F. G. Barth, J. Comp. Physiol. **1985**, 157, 115.

[11] F. G. Barth, Z. Zellforsch. Mikrosk. Anat. **1973**, 144, 409.

[12] F. Song, K. W. Xiao, K. Bai, Y. L. Bai, Mater. Sci. Eng. A **2007**, 457, 254.

[13] S. Kilper, S. J. Facey, Z. Burghard, B. Hauer, D. Rothenstein, J. Bill, Adv. Funct. Mater. **2018**, 28, 1705842.
